# Effects of Intranasal Oxytocin on the Interpretation and Expression of Emotions in Anorexia Nervosa

**DOI:** 10.1111/jne.12458

**Published:** 2017-03-08

**Authors:** J. Leppanen, V. Cardi, K. W. Ng, Y. Paloyelis, D. Stein, K. Tchanturia, J. Treasure

**Affiliations:** ^1^ Department of Psychological MedicineInstitute of Psychiatry, Psychology, and NeuroscienceKing's College LondonLondonUK; ^2^ Singapore General HospitalSingaporeSingapore; ^3^ Division of NeuroscienceDepartment of NeuroimagingThe Institute of Psychiatry, Psychology and NeuroscienceKing's College LondonLondonUK; ^4^ Chaim Sheba Medical CenterEdmond and Lily Safra Children's HospitalTel HashomerIsrael; ^5^ Department of PsychologyIlia State UniversityTbilisiGAUSA

**Keywords:** oxytocin, anorexia nervosa, social‐emotional functioning, facial expressions, positive affect, negative affect

## Abstract

Altered social‐emotional functioning is considered to play an important role in the development and maintenance of anorexia nervosa (AN). Recently, there has been increasing interest in investigating the role of intranasal oxytocin in social‐emotional processing. The present study aimed to investigate the effects of intranasal oxytocin on the interpretation and expression of emotions among people with AN. Thirty women with AN and 29 age‐matched healthy women took part in the present study, which used a double‐blind, placebo‐controlled, cross‐over design. The participants received a single dose of 40 IU of intranasal oxytocin in one session and a placebo spray in the other. Fifteen minutes after administration, the participants completed the Reading the Mind in the Eyes Test to assess the interpretation of complex emotions and mental states followed by a video task, which assessed expressions of facial affect when they were viewing humorous and sad film clips. The intranasal oxytocin did not significantly influence the expression or interpretation of emotions in the AN or healthy comparison groups. The AN group expressed significantly less positive emotion, spent more time looking away and reported experiencing a significantly more negative affect in response to the film clips. The finding that intranasal oxytocin had little to no effect on the interpretation or expression of emotions in either group supports the notion that the effects of oxytocin on social‐emotional processing are not straightforward and may depend on individual and environmental differences, as well as the emotion being processed. Replication of these findings is necessary to explore the effect of timing on the effects of oxytocin before firm conclusions can be drawn. Nonetheless, these findings add to the steady accumulation of evidence that people with AN have reduced emotional expression and avoidance of emotionally provoking stimuli.

Current models of anorexia nervosa (AN) postulate that altered social cognition and difficulties in social‐emotional functioning contribute to the development and maintenance of AN 1, 2. Robust evidence from recent meta‐analyses shows that people with AN have difficulties in a wide range of social‐emotional domains, including the accurate interpretation and appropriate expression of emotions. Appropriate facial expression and mimicry increase prosocial behaviour and aid the accurate interpretation of emotions, whereas an inappropriate or blunted expression of emotions has been found to give rise to negative judgements and elicit a desire for increased social distance 3, 4, 5. In eating disorders, it has been suggested that problems in social communication contribute to the maintenance of disordered eating by causing interpersonal difficulties and increasing isolation 1, 2. Thus, treatments targeting these interpersonal problems may be beneficial for people with AN.

Behavioural studies have demonstrated that people with AN have particular difficulties with accurately interpreting positive and negative emotions in faces, as well as tone of voice 6. People with AN also have difficulties with accurately interpreting complex emotions and mental states in tasks such as the Reading the Mind in the Eyes Test (RMET) and other theory of mind tasks 6. Furthermore, a recent meta‐analysis showed that people with AN, depression and autism display fewer positive and negative facial expressions when viewing positive and negative emotional film clips 7. Interestingly, people who have recovered from AN do not show similar difficulties in the interpretation and expression of emotions 6, 7, 8, 9. Thus, these difficulties are possible secondary consequences of malnutrition; however, the exact mechanisms underlying difficulties in social‐emotional processing in AN are still unclear.

The neuropeptide oxytocin is considered to pay a key role in social‐emotional functioning 10, 11. A recent review has documented a range of abnormalities in oxytocin functioning in acute AN 12. Additionally, another recent meta‐analysis found that people with AN have significantly lower peripheral endogenous oxytocin levels than healthy individuals 13. Moreover, preliminary proof of concept studies have found that a single dose of intranasal oxytocin influences certain aspects of social‐emotional processing in eating disorders 14, 15. Among people with AN, a single dose of intranasal oxytocin attenuated attentional bias towards disgusted faces 15, although it did not increase sensitivity to recognise basic emotions, whereas, in people with bulimia nervosa and healthy comparison (HC) participants, intranasal oxytocin increased sensitivity to detect emotions 14. These mixed findings suggest that further exploration of the effects of a single dose of intranasal oxytocin on social‐emotional functioning is still necessary.

A single dose of intranasal oxytocin has also been reported to improve the interpretation of complex emotional states in a number of different paradigms, including the RMET and the awareness of social inference test, among healthy individuals 16, 17, 18. Additionally, a single dose has been reported to further improve the interpretation of complex emotions among those healthy individuals who report difficulties in identifying and describing emotions 19. Among clinical populations, a single dose of intranasal oxytocin has been reported to improve the interpretation of complex emotions among people with schizophrenia and depression, comprising disorders characterised by difficulties in the interpretation of emotions 16, 20. Thus, these findings suggest that a single dose of intranasal oxytocin may increase attention social‐emotional cues particularly among those individuals who have difficulties with such social‐emotional processes. However, to our knowledge, no studies to date have investigated the effects of a single dose of intranasal oxytocin on the interpretation of complex emotions in AN.

A single dose of intranasal oxytocin has also been found to increase emotional empathy and cooperation among healthy individuals particularly when social information regarding the other person was present 21, 22. Additionally, recent studies have found that a single dose of intranasal oxytocin can increase cooperation and normalise the avoidant, flattened arousal responses to human social sounds in autism spectrum disorder 23, 24. Thus, oxytocin may be a facilitator of social communication, including facial expression of emotion. Few studies thus far have investigated the effects of a single dose of oxytocin on emotion expression 25, 26. Among healthy populations, a single dose of intranasal oxytocin has been reported to increase spontaneous facial mimicry in response to emotionally provoking videos, as well as increase facial expressivity during the instructed production of facial expressions 25, 26. Among clinical populations, a single dose of intranasal oxytocin has been reported to increase facial expressivity in response to emotionally provoking images among people with schizophrenia and to reduce avoidant behaviours during a clinical interview in people with borderline personality disorder 27, 28. However, to our knowledge, no studies to date have investigated the effects of intranasal oxytocin on evoked emotional facial expressions and the interpretation of complex emotions in AN.

The present study aimed to investigate the effects of a single dose of intranasal oxytocin on the interpretation and expression of emotions in people with AN and healthy control (HC) participants. Based on the previous work outlined above, we hypothesised that participants with AN would have anomalies in the interpretation and expression of emotions and that oxytocin administration would alleviate these anomalies. Additionally, we explored correlations between oxytocin‐induced changes in the interpretation and expression of emotions and depression, autistic traits, and body mass index (BMI) within the AN group. Finally, we also explored differences in the interpretation and expression of emotions, as well as the effects of oxytocin, between AN participants who were taking antidepressants during the study and those AN participants who were free of psychotropic medication.

## Materials and methods

### Participants

Thirty women with AN were recruited to take part in the present study. The AN participants were recruited through South London and Maudsley National Health Service (NHS) Trust and eating disorder charity websites (BEAT https://www.b-eat.co.uk/ and Succeed www.succeed-foundation.org). All interested AN participants were included in the study if they were female, aged 16–65 years, met the DSM‐5 criteria for AN assessed with the Structured Clinical Interview for DSM‐5 29 and did not meet any of the exclusion criteria for all participants. Fifteen of the AN participants were receiving psychotropic medication during the study. Twenty‐nine HC women were recruited to take part in the study as a comparison group. The HC participants were recruited through King's College London circulars and notice boards in the local community. All HC participants were screened for past and current psychiatric disorders with the Structured Clinical Interview for DSM‐5 29 and excluded if they reported any current or history of mental health difficulties or suicidality. Additionally, participants were excluded if they met any of the overall exclusion criteria for all participants, which included being pregnant, a history of (or current) alcohol or drug abuse/misuse, hormonal disturbance not as a result of low weight among the AN participants, current impairments in cardiovascular functioning, and any regular medication excluding psychotropic medication among the AN participants. Five HC participants reported taking the contraceptive pill and were asked to take a break from the pill and wait for the onset of their next menstrual cycle before taking part. Prior to taking part in the study, all participants provided their written, informed consent. The study was approved by the National Health System Research Ethics Committee (14/LO/0128) and was conducted in accordance with the Helsinki Declaration of 1975 (revised in 2008).

The sample size was based on an *a priori* power analysis conducted with G*Power, which indicated that, altogether, 60 participants would be needed to have sufficient statistical power (0.8) to detect significant effects 30.

### Self‐report measures

The Eating Disorder Examination Questionnaire (EDEQ) was used to obtain an assessment of eating disorder psychopathology with the subscales: restraint, eating concern, weight concern and shape concern 31. The reliability of the EDEQ was high in the present study (Cronbach's *α *= 0.98).

Assessments of depression, anxiety and stress were obtained using the Depression, Anxiety and Stress Scale (DASS) 32. The reliability of the DASS was high in the present study (Cronbach's *α *= 0.98).

Autism Spectrum Quotient (AQ‐10) is a short 10‐item assessment of autistic traits with high positive predictive value at clinical cut‐off of 6 33. The reliability of the AQ‐10 was acceptable in the present study (Cronbach's *α *= 0.70).

### Design and procedure

The present study used a within subjects, placebo‐controlled, double‐blind AB/BA cross‐over design to explore the effects of intranasal oxytocin on evoked facial expressions in AN. The study consisted of two sessions. In one session, participants were asked to self‐administer 10 puffs (five in each nostril) of a nasal spray containing 40 IU of synthetic oxytocin (Syntocinon; Novartis Pharmaceutical, Basel, Switzerland). In the other session, participants were asked to self‐administer 10 puffs (five in each nostril) of a placebo (placebo) nasal spray. The dose was based on previous work investigating the effects of a single dose of intranasal oxytocin in eating disorders 14, 15, 34, 35. The placebo nasal spray was custom made to be identical to the oxytocin spray minus the active ingredient. The order in which participants received oxytocin and placebo was counterbalanced and randomised. All HCs, and AN participants if menstruating, were tested during the first 1–10 days of the menstrual cycle. Additionally, the participants were asked not to consume any food 2 h before the sessions and to refrain from smoking or drinking alcoholic or caffeinated drinks 12 h before the sessions.

Fifteen minutes after administration, the participants were asked to complete a computerised version of the RMET 36 presented with eprime, version 2.0 (Psychology Software Tools, Inc., Sharpsburg, PA, USA). The RMET consists of a short practice trial and an experimental trial. In the practice trial, participants were presented with 10 images of eyes and were asked to identify whether the eyes were male or female. In the experimental trial, participants are presented 36 pictures of eyes (18 male and 18 female) depicting complex emotions. In this part of the task, participants are asked to identify the emotion that the eyes are conveying by selecting one of four options presented in each corner of the screen. The pictures were presented in a randomised order in each session. In the present study, participants were given infinite time to respond to each trial before moving onto the next. Participants' reaction times and accuracy were recorded.

Twenty‐five minutes after administration, the participants were asked to watch two short video clips, each lasting 2–2.5 min, during which they were filmed. The two clips were chosen to evoke positive and negative emotions in the participants. The first film clip (henceforth Film 1) was a humorous scene from the film *Four Weddings and a Funeral* (1994), which has been previously successfully been used in a number of studies to evoke positive emotions 9, 37, 38, 39. The second film clip (henceforth Film 2) was a sad scene chosen from the film *Shadowlands* (1999), which has also been previously successfully used to evoke negative emotions in the studies listed above 9, 37, 38, 39. The same film clips were presented in both sessions in a fixed order with a short clip of computer simulated waves presented before each clip. This was performed to avoid carry‐over effects from emotions experienced before the task started, as well as to avoid carry‐over effects from the first film clip onto the second film clip.

After viewing each clip, participants were asked to rate how they were feeling on the Positive and Negative Affect Schedule (PANAS) 40. The PANAS consist of 20 emotion words and participants were asked to rate to what extent they were experiencing each emotion on a scale of 1 (very slightly or not at all) to 5 (extremely). The PANAS ratings were then analysed to establish participants self‐reported positive and negative affect scores in response to Film 1 and Film 2.

### Noldus facereader

Noldus facereader (Noldus Information Technology BV, Wageningen, The Netherlands) comprises facial expression analysis software that has been developed to identify six basic emotions: happiness, sadness, fear, anger, surprise and disgust. The software also detects neutral expressions. facereader reports the intensity to which each of the six basic emotions are expressed in each frame on a scale of 0 to 1, where 0 indicates that the emotion is not present and 1 indicates that the emotion is fully present. facereader has been validated using manual emotion coding systems and facial electromyography 41, 42, 43. The present study focused on exploring expressions of happiness and sadness.

### Looking away

Looking away was analysed manually. The total number of seconds participants looked away from the film stimuli was recorded and analysed.

### Statistical analysis

All data were analysed using stata, version 14 (StataCorp, College Station, TX, USA). Differences in clinical and demographic self‐report measures between the groups were analysed using a nonparametric median chi‐squared test.

As a result of the highly skewed nature of the data, the PANAS ratings were transformed with square root transformation prior to analysis. Group differences and effects of oxytocin/placebo on the expression of emotions, PANAS ratings and looking away were explored with bootstrapped mixed linear models with 1000 bootstrap replications. Drug (oxytocin, placebo), Film (Film 1, Film 2), Group (AN, HC) and session (session 1, session 2) were entered as fixed effects with a random intercept. Technical difficulties with the video camera SD card led to loss of data from one AN participant and three HC participants.

As described above, group differences and the effect of drug on the interpretation of emotions on the RMET were examined using a bootstrapped mixed linear model (1000 replications) with Drug (oxytocin, placebo), Group (AN, HC) and Session (session 1, session 2) entered as fixed effects and a random intercept. As a result of the close proximity of the sessions (1–5 days apart), we included Session as a fixed effect to control for familiarity with the RMET and film stimuli. Significant interactions were further explored by investigating post‐hoc contrasts and pairwise comparisons. We additionally conducted further post‐hoc analysis investigating the effects of oxytocin on easy and difficult items between the two groups. We used a median split to divide items into easy (accuracy > 75.42%) and difficult (accuracy < 75.42%) groups based on total sample accuracy. These results are presented in the Supporting information (Doc S1, Table S2).

Additionally, we explored differences in the effects of intranasal oxytocin between AN participants taking antidepressants during the time of the study and those free of psychotropic medication in the above measures. As above, the effects of drug on the interpretation and expression of emotions, PANAS ratings and looking away were explored with similar 2 × 2 × 2 × 2 and 2 × 2 × 2 bootstrapped mixed linear models with 1000 bootstrap replications. The results are presented in the Supporting information (Doc. S1, Table [Link], [Link], [Link], [Link], Figure [Link], [Link]).

Within the AN group, relationships between oxytocin‐induced changes in the interpretation and expression of emotions, and depression (DASS: depression subscale), autistic features (AQ‐10) and BMI were analysed with Spearman's rho correlation. Prior to correlation analysis, oxytocin‐induced changes in the interpretation and expression of emotions were calculated by subtracting the interpretation accuracy and facial expression scores in the placebo condition from those in the oxytocin condition. Thus, positive scores indicated greater accuracy and facial expressivity in the oxytocin condition, whereas negative scores indicated greater accuracy and facial expressivity in the placebo session. The results are presented in the Supporting information (Doc S1, Table S6).

## Results

### Clinical and demographic characteristics

The clinical characteristics of the sample are presented in Table 1. The AN and HC groups were matched for age. The AN group had a significantly lower BMI than the HC group. Additionally, the AN group a reported significantly higher incidence of eating disorder psychopathology as measured on the EDEQ, and reported higher restraint, eating concern, shape concern and weight concern. Furthermore, the AN group scored higher on the DASS, reporting more depression, anxiety and stress than the HC group. Finally, there was a significant difference between the two groups on level of education, with HC participants reporting a higher level of education than the AN participants, 90% of whom reported having taken time off from education as a result of their illness.

**Table 1:** Clinical and Demographic Sample Characteristics

	AN (n = 30)	Medicated AN (n = 15)	Non‐medicated AN (n = 15)	Medicated AN versus non‐medicated AN	HC (n = 29)	AN versus HC
	Median (Q1, Q3)	Median (Q1, Q3)	Median (Q1, Q3)	χ^2^, P value	Median (Q1, Q3)	χ^2^, P value
BMI	16.13 (14.56, 18.14)	17.63 (15.20, 18.51)	14.67 (14.37, 16.61)	2.13, 0.144	22.21 (20.78, 25.51)	35.55, < 0.001
Age	24.50 (22.00, 28.25)	25.00 (22.00, 27.00)	24.00 (21.00, 29.00)	0.00, 1.000	25.00 (23.00, 27.50)	0.02, 0.875
Level of education	3.00 (1.00, 3.00)	3.00 (1.00, 3.00)	3.00 (1.00, 3.50)	0.00, 1.000	3.00 (3.00, 4.00)	12.34, < 0.001
Time off education as a result of AN (years)	1.00 (0.69, 2.00)	1.50 (0.92, 2.50)	0.67 (0.25, 0.1.25)	2.17, 0.141	–	–
EDEQ total	4.13 (3.00, 5.13)	4.49 (4.21, 5.50)	3.68 (2.66, 4.06)	19.27, < 0.001	0.59 (0.34, 1.00)	35.27, < 0.001
EDEQ restraint	3.70 (2.60, 5.05)	4.40 (2.60, 5.20)	3.40 (1.40, 4.80)	0.07, 0.796	0.60 (0.00, 1.70)	20.79, < 0.001
EDEQ weight concern	4.20 (2.70, 5.65)	5.60 (3.80, 6.00)	3.00 (2.00, 4.60)	5.40, 0.020	0.60 (0.30, 1.40)	31.38, < 0.001
EDEQ shape concern	4.86 (3.56, 5.91)	5.88 (5.38, 6.00)	4.25 (2.75, 4.75)	19.27, < 0.001	0.75 (0.38, 1.38)	41.67, < 0.001
EDEQ eating concern	4.10 (2.90, 4.80)	4.40 (3.20, 4.80)	3.6 (2.60, 4.40)	5.40, 0.020	0.20 (0.00, 0.40)	41.67, < 0.001
DASS Total	63.00 (40.00, 86.00)	72.00 (54.00, 88.00)	46.00 (26.00, 86.00)	6.67, 0.010	4.00 (1.00, 10.00)	37.49, < 0.001
DASS Depression	21.00 (13.50, 32.00)	30.00 (20.00, 40.00)	18.00 (10.00, 26.00)	6.67, 0.010	0.00 (0.00, 2.00)	26.61, < 0.001
DASS Anxiety	12.00 (6.00, 21.00)	18.00 (10.00, 26.00)	8.00 (2.00, 20.00)	4.29, 0.038	0.00 (0.00, 2.00)	29.07, < 0.001
DASS Stress	25.00 (17.50, 31.00)	26.00 (24.00, 30.00)	18.00 (14.00, 34.00)	0.27, 0.606	2.00 (0.00, 7.00)	37.49, < 0.001
AQ	3.00 (4.00, 1.00)	2.00 (4.00, 1.00)	3.00 (4.00, 1.00)	0.17, 0.680	2.00 (3.00, 1.00)	1.38, 0.502

AN, anorexia nervosa; HC, healthy comparison; BMI, body mass index; EDEQ, Eating Disorder Examination Questionnaire; DASS, Depression, Anxiety and Stress Scale; AQ, Autism quotient; Level of education: 1 = A‐level/National Vocational Qualification, 2 = Diploma, 3 = Undergraduate degree, 4 = Postgraduate degree.

There were no significant differences in age or BMI between those AN participants taking antidepressants during the study and those AN participants who were free of psychotropic medication (Table 1). The medicated AN participants did report a higher incidence of eating disorder symptomatology as measured on the EDEQ, scoring higher on the eating concern, weight concern and shape concern subscales than those free of medication. Additionally, the medicated AN participants scored higher on the DASS, reporting more depression and anxiety but not stress than the non‐medicated AN participants (Table 1).

### Performance on the RMET

**Figure 1:**
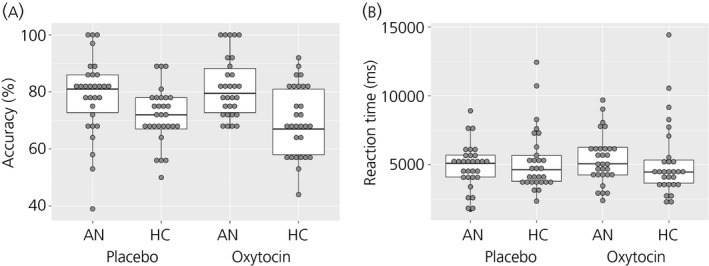
Percentage accuracy (a) and reaction times (b) on the Reading the Mind in the Eyes (RMET) in the anorexia nervosa (AN) and healthy comparison (HC) groups in the oxytocin and placebo conditions. Grey dots represent individual data points; the box plot represents the median, interquartile range, and the maximum and minimum of the data. RT, reaction time; ms, milliseconds.

Interpretation accuracy and reaction times (RT) in the AN and HC groups following both oxytocin and placebo administration are presented in Fig. 1(A,B). The mixed model revealed a significant difference between groups, with the AN participants being significantly more accurate than the HC participants (Table 2). There was also a significant effect of session, with all participants being significantly more accurate in session 2 than session 1 [Z = 2.39, P = 0.017, 95% confidence interval (CI) = 0.01–0.12]. There were no other significant effects or interactions influencing accuracy.

**Table 2:** Performance on the Reading the Mind in the Eyes Test (RMET) in Anorexia Nervosa (AN) and Healthy Comparison (HC) Groups Following Oxytocin and Placebo

	Drug	AN (n = 30), mean ± SD	HC (n = 29), mean ± SD	χ^2^, P value
Accuracy (%)	Oxytocin	81.77 ± 10.67	69.83 ± 12.35	Drug: 0.27, 0.604 Session: 5.69, 0.017 Group: 31.25, < 0.001 Drug × Session: 2.86, 0.091 Drug × Group: 1.33, 0.250 Session × Group: 0.71, 0.400 Drug × Session × Group: 0.72, 0.397
	Placebo	78.83 ± 13.74	71.79 ± 9.63	
RT	Oxytocin	5379.72 ± 1802.67	5158.24 ± 2671.37	Drug: 0.65, 0.420 Session: 14.90, < 0.001 Group: 0.01, 0.909 Drug × Session: 0.97, 0.324 Drug × Group: 1.84, 0.175 Session × Group: 0.01, 0.920 Drug × Session × Group: 11.31, 0.001
	Placebo	4929.32 ± 1600.89	5241.59 ± 2282.45	

RT, reaction time.

In terms of RT, the mixed model revealed a significant effect of session, with all participants responding faster in session 2 than session 1 (Z = −3.86, P < 0.001, 95% CI = −1211.80 to −395.67). There was also a significant Drug × Group × Session interaction (Table 2). The interaction was first explored by investigating the Drug × Group interaction within each session. The results revealed that there was a significant Drug × Group interaction in session 2 (χ^2^ = 12.53, P = 0.0004) but not in session 1 (χ^2^ = 2.70, P = 0.1006). The effect of drug was then investigated within each group in session 2. The results revealed that oxytocin led to a significantly slower RT in the AN group in (Z = 2.32, P = 0.020, 95% CI = 157.26–1871.20) and a faster RT in the HC group in session 2 (Z = −2.62, P = 0.009, 95% CI = −2059.48 to −269.17).

We also explored the Group × Session within placebo and oxytocin conditions. The post‐hoc contrasts revealed a significant Group × Session interaction following both placebo (χ^2 ^= 6.83, P = 0.009) and oxytocin administration (χ^2^ = 6.49, P = 0.011). The pairwise comparisons revealed that HC participants were significantly faster than the AN participants in session 2 (Z = −2.59, P = 0.010, 95% CI = −1912.21 to −263.49) but not in session 1 (Z = 1.13, P = 0.259, 95% CI = −431.42 to 1602.88) following oxytocin administration. Following placebo administration, the HC participants were significantly slower than the AN participants in session 2 (Z = 2.45, P = 0.014, 95% CI = 222.32–1986.10) but not in session 1 (Z = −1.19, P = 0.235, 95% CI = −1293.64–317.61) (Table 2).

Because 17 of the AN participants had completed the RMET before, we also explored differences between the groups on task performance without these participants to examine their impact. These findings are presented in the Supporting information (Doc S1). The results revealed that, although the AN participants were still on average more accurate than the HC participants, this difference no longer reached significance (see Supporting information, Table S1). As above, there was a significant effect of session suggesting that participants were more accurate in session 1 than in session 2.

Regarding RT, the results revealed a significant effect of session, with all participants being faster in session 2, and a significant Drug × Group interaction (see Supporting information, Table S1). Further exploration of the interaction revealed that the AN participants who had not completed the RMET before were significantly slower in the oxytocin than placebo condition (Z = 2.70, P = 0.007, 95% CI = 165.63–1045.27). A similar effect was not present in the HC group (Z = −0.56, P = 0.576, 95% CI = −503.51 to 280.00).

### Expressions of happiness and sadness, and looking away

**Figure 2:**
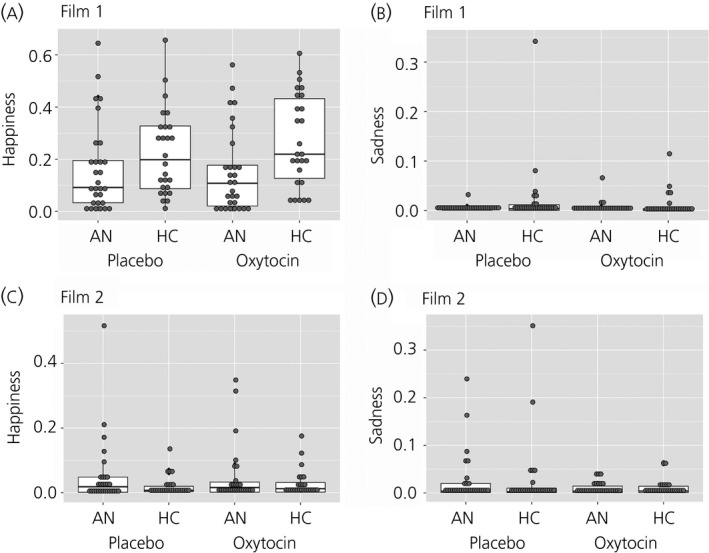
Intensity of expressions of sadness and happiness in response to Film 1 and Film 2 in the anorexia nervosa (AN) and healthy comparison (HC) groups in oxytocin and placebo conditions. (a) Expressions of happiness in response Film 1, (b) expressions of sadness in response to Film 1, (c) expressions of happiness in response to Film 2, (d) expressions of sadness in response to Film 2. Grey dots represent individual data points; the box plot represents the median, interquartile range, and the maximum and minimum of the data. Film 1, humorous film clip; Film 2, sad film clip.

The intensity of expression of sadness and happiness in response to Film 1 and Film 2 following placebo and oxytocin administration is summarised for the AN and HC groups in Fig. 2(a,b) for Film 1 and Fig. 2(c,d) for Film 2. The P‐value was adjusted for multiple post‐hoc comparisons using the false discovery rate and was set at P < 0.003 44. As expected, the mixed model revealed a significant effect of Film, with all participants across groups and sessions expressing more happiness when viewing Film 1 than Film 2. The results also revealed a significant effect of Group, with HC participants expressing more positive facial expressions than AN participants across films and sessions (Table 3). The model also revealed a significant Film × Group and Drug × Film × Group × Session interactions.

**Table 3:** Expressions of Happiness and Sadness, and Looking Away in Anorexia Nervosa (AN) and Healthy Comparison (HC) Groups During Film 1 and Film 2

	Film	Drug	AN (n = 30), mean ± SD	HC (n = 29), mean ± SD	χ^2^, P value
Expressions of happiness	Film 1	Oxytocin	0.15 ± 0.16	0.27 ± 0.18	Drug: 0.47, 0.494 Film: 134.66, < 0.001 Group: 5.81, 0.016 Session: 0.20, 0.592 Drug × Film: 0.12, 0.727 Drug × Group: 1.63, 0.202 Film × Group: 14.29, < 0.001 Drug × Session: 0.05, 0.818 Film × Session: 2.08, 0.149 Group × Session: 1.11, 0.293 Drug × Film × Group: 0.29, 0.592 Drug × Film × Session: 0.03, 0.857 Drug × Group × Session: 0.94, 0.857 Film × Group × Session: 0.01, 0.905 Drug × Film × Group × Session: 4.07, 0.044
		Placebo	0.16 ± 0.17	0.22 ± 0.16	
	Film 2	Oxytocin	0.05 ± 0.09	0.03 ± 0.04	
		Placebo	0.05 ± 0.10	0.02 ± 0.03	
Expressions of sadness	Film 1	Oxytocin	0.01 ± 0.01	0.01 ± 0.02	Drug: 3.62, 0.057 Film: 7.19, 0.007 Group: 0.61, 0.436 Session: 1.73, 0.188 Drug × Film: 0.76, 0.384 Drug × Group: 0.73, 0.392 Film × Group: 1.30, 0.254 Drug × Session: 0.06, 0.801 Film × Session: 0.57, 0.451 Group × Session: 0.34, 0.559 Drug × Film × Group: 1.13, 0.289 Drug × Film × Session: 0.64, 0.422 Drug × Group × Session: 0.71, 0.398 Film × Group × Session: 0.23, 0.631 Drug × Film × Group × Session: 0.48, 0.488
		Placebo	0.004 ± 0.006	0.02 ± 0.07	
	Film 2	Oxytocin	0.01 ± 0.01	0.01 ± 0.02	
		Placebo	0.03 ± 0.05	0.03 ± 0.08	
Looking away (s)	Film 1	Oxytocin	1.28 ± 2.49	0.23 ± 0.51	Drug: < 0.01, 0.948 Film: 8.58, 0.003 Group: 18.05, < 0.001 Session: 7.97, 0.005 Drug × Film: 0.07, 0.797 Drug × Group: 0.72, 0.397 Film × Group: 1.87, 0.172 Drug × Session: 3.11, 0.078 Film × Session: 0.09, 0.762 Group × Session: 3.31, 0.069 Drug × Film × Group: 0.12, 0.728 Drug × Film × Session: 0.09, 0.767 Drug × Group × Session: 1.98, 0.160 Film × Group × Session: 0.04, 0.841 Drug × Film × Group × Session: 2.59, 0.107
		Placebo	1.00 ± 1.93	0.31 ± 0.74	
	Film 2	Oxytocin	2.72 ± 3.72	0.65 ± 2.08	
		Placebo	2.38 ± 4.69	1.08 ± 2.35	

Film 1, humorous film clip; Film 2, sad film clip.

The Film × Group interaction was investigated further by exploring the difference between the groups at the two levels of Film. The post‐hoc test revealed that the HC participants expressed significantly more happiness than the AN participants when viewing Film 1 (Z = 4.29, P < 0.0001, 95% CI = 0.08–0.21). There were no significant differences between the groups in expressions of happiness when viewing Film 2 (Z = −1.34, P = 0.181, 95% CI = −0.10–0.02).

The four‐way interaction was first investigated by exploring the Drug × Film × Group interaction within session 1 (χ^2^ = 3.84, P = 0.05) and session 2 (χ^2^ = 1.02, P = 0.314), which did not yield significant results. Next, the Drug × Film × Session interaction was explored within the HC (χ^2^ = 2.23, P = 0.135) and AN groups (χ^2^ = 1.85, P = 0.173), which did not yield significant results. Finally, the Drug × Session × Group interaction was explored within Film 1 (χ^2^ = 4.06, P = 0.044) and Film 2 (χ^2^ = 1.07, P = 0.301), which also did not yield significant results. There were no other significant effects or interactions influencing expressions of happiness.

Regarding the expression of sadness, as above, the mixed model revealed a significant effect of Film, with all participants across groups and sessions expressing more sadness when viewing Film 2 than Film 1. There were no other significant effects or interactions influencing expressions of sadness.

The total time (s) that participants looked away from the film stimuli is presented in Table 3. The mixed model revealed a significant effect of group, with AN participants looking away more than HC participants. There was also a significant effect of film and session, with all participants spending more time looking away from Film 2 than Film 1, and looking away more during session 2 than session 1. There were no other significant effects or interactions (Table 3).

### Subjective PANAS ratings

Ratings of subjective positive and negative affect following Film 1 and Film 2 in the AN and HC groups are presented in Table 4. The P‐value was adjusted for multiple post‐hoc comparisons using the false discovery rate and was set at P < 0.004 44. The mixed model exploring ratings of positive affect on the PANAS revealed a significant effect of group in ratings of positive affect, with the HC participants reporting a more subjective positive affect relative to the AN participants (Table 4). There was also a significant effect of Film, with all participants reporting more positive affects following Film 1 than Film 2, and a significant effect of session, with participants reporting a more positive affect in session 1 than session 2.

**Table 4:** Positive and Negative Affect Schedule (PANAS) Rating Following Film 1 and Film 2 in Anorexia Nervosa (AN) and Healthy Comparison (HC) Groups

PANAS	Film	Drug	AN (n = 30), mean ± SD	HC (n = 29), mean ± SD	χ^2^, P value
Positive affect	Film 1	Oxytocin	11.00 ± 4.67	14.36 ± 7.16	Drug: 1.30, 0.254 Film: 131.20, < 0.001 Group: 58.09,< 0.001 Session: 10.66, 0.001 Drug × Film: 0.05, 0.829 Drug × Group: 0.29, 0.587 Film × Group: 0.02, 0.879 Drug × Session: 1.09, 0.297 Film × Session: 6.38, 0.012 Group × Session: 4.10, 0.043 Drug × Film × Group: 0.07, 0.789 Drug × Film × Session: 4.54, 0.032 Drug × Group × Session: 0.02, 0.882 Film × Group × Session: 2.53, 0.111 Drug × Film × Group × Session: 0.43, 0.512
		Placebo	11.72 ± 5.71	15.84 ± 6.04	
	Film 2	Oxytocin	5.21 ± 4.29	8.72 ± 7.27	
		Placebo	5.57 ± 4.61	9.60 ± 6.51	
Negative affect	Film 1	Oxytocin	5.07 ± 6.08	0.88 ± 1.69	Drug: 0.64, 0.425 Film: 33.10, < 0.001 Group: 69.99, < 0.001 Session: 0.05, 0.824 Drug × Film: < 0.01, 0.950 Drug × Group: 0.05, 0.820 Film × Group: 1.32, 0.251 Drug × Session: 1.14, 0.285 Film × Session: 0.29, 0.592 Group × Session: 0.41, 0.521 Drug × Film × Group: 0.28, 0.599 Drug × Film × Session: 0.10, 0.750 Drug × Group × Session: 1.54, 0.215 Film × Group × Session: 0.03, 0.872 Drug × Film × Group × Session: 0.18, 0.673
		Placebo	4.28 ± 4.57	0.88 ± 1.45	
	Film 2	Oxytocin	8.82 ± 6.78	3.68 ± 5.37	
		Placebo	8.57 ± 5.49	3.08 ± 3.30	

Film 1, humorous film clip; Film 2, sad film clip.

The mixed model exploring ratings of subjective positive affect also revealed significant Film × Session and Drug × Film × Session interactions (Table 4). The three‐way interaction was first explored by investigating the Drug × Film interaction in session 1 and session 2, which revealed no significant interactions (session 1: χ^2^ = 2.53, P = 0.112; session 2: χ^2^ = 4.04, P = 0.044). The three‐way interaction was then explored by investigating the Session × Drug interaction in the two levels of film, which revealed no significant interactions (Film 1: χ^2^ = 5.24, P = 0.023; Film 2: χ^2^ = 1.34, P = 0.247). Finally, the three‐way interaction was explored by investigating the Film × Session interaction at the two levels of drug, which revealed a significant interaction in the oxytocin (χ^2^ = 11.19, P = 0.001) but not placebo session (χ^2^ < 0.01, P = 0.980). Further pairwise comparison revealed that participants who received oxytocin in the first session and those who received it in the second session reported a more subjective positive affect in response to Film 1 than Film 2 (session 1; Z = −7.55, P < 0.001, 95% CI = −11.42 to −6.71; session 2: Z = −2.16, P = 0.031, 95% CI = −5.00 to −0.24). There was also a significant Group × Session interaction (Table 4), with HC participants reporting a significantly more positive affect in session 1 than 2 (Z = −3.84, P < 0.001, 95% CI = −4.21 to −1.36). There was no significant differences between the session within the AN group (Z = −0.84, P = 0.399, 95% CI = −2.14 to 0.86).

The mixed model exploring ratings of negative affect revealed a significant effect of Film, with all participants reporting significantly more negative affect in response to Film 2 than Film 1. There was also a significant difference between groups in ratings of negative affect, with AN participants reporting a more negative affect than the HC participants (Table 4).

## Discussion

The present study aimed to investigate the effects of a single dose of intranasal oxytocin on the interpretation and expression of emotions in people with AN and HC participants. By contrast to what was hypothesised based on the previous literature, oxytocin administration did not have a significant impact on the interpretation or expression of emotions in either group. However, as hypothesised, the present findings replicated previous work demonstrating that, relative to HC, people with AN display a less positive facial affect, spend more time looking away from the emotional stimuli, and report a less subjective positive affect when viewing emotional film stimuli. Surprisingly, the AN participants were significantly more accurate with respect to interpreting complex emotions in the RMET than the HC participants. Finally, we found that AN participants who were taking antidepressants during the study expressed a less positive facial affect when viewing the humorous film and were less accurate with respect to interpreting complex emotions relative to non‐medicated AN participants.

By contrast to what was hypothesised, we did not find significant effects of intranasal oxytocin on the expression of facial affect. These findings were somewhat surprising considering that previous studies have reported a significant effect of oxytocin on expression of emotions among healthy and clinical populations 25, 26, 27. The discrepancy could arise from the fact that previous studies have largely utilised different kinds of emotionally provoking stimuli, including threatening social and nonsocial stimuli 25, 26, 27. Indeed, Korb and Malsert 25 found no changes in the expression of facial affect in response to positive facial expressions, although they did find changes in frowning in response to angry faces. Furthermore, intranasal oxytocin may differentially influence spontaneous facial expression, instructed production of facial expression 26 and facial mimicry during an emotion identification task 25. However, in a recent meta‐analysis and systematic review conducted by our group, we found that oxytocin did not have a significant effect on emotion expression (J. Leppanen, K. W. Ng, K. Tchanturia and J. Treasure, unpublished data).

Additionally, we found no significant effects of oxytocin administration on the accuracy of complex emotion interpretation in the AN or HC groups. In part, these findings support a recent proof of concept study, which found that intranasal oxytocin did not improve sensitivity to recognise basic emotions in AN 14. However, the previous study used a different task to assess interpretation of emotions and found that intranasal oxytocin improved emotion recognition sensitivity in HC participants and people with bulimia nervosa 14. Similarly, other previous studies have reported that a single dose of intranasal oxytocin significantly improved recognition of complex emotions on the RMET among healthy individuals 17, 18. However, recently, there have also been some failed replications of these findings suggesting that the effects of intranasal oxytocin on social‐emotional processing are likely to more complex than previously anticipated 45, 46, 47. Furthermore, in our recent meta‐analysis and systematic review, we found that, when fourteen previous studies were pooled together, a single dose on intranasal oxytocin did not have a significant impact on any theory of mind measures, including RMET (J. Leppanen, K. W. Ng, K. Tchanturia and J. Treasure, unpublished data). However, in a simpler test of recognition of basic emotions, a single dose of oxytocin improved recognition of fear and increased sensitivity to recognise anger in healthy individuals only (J. Leppanen, K. W. Ng, K. Tchanturia and J. Treasure, unpublished data).

Indeed, a systematic review has reported that almost half of studies investigating the effects of intranasal oxytocin on social‐emotional processing did not report significant effects of oxytocin. Furthermore, where significant effects were found, 63% of the time, the effects were moderated by environmental factors or individual differences 10. Additionally, a more recent systematic review has suggested that the varied effects of oxytocin on social‐emotional processing may be moderated by social boundaries, such that oxytocin not only increases social‐affiliative behaviour within an ‘in‐group’, but also increases distrust and desire to punish strangers and ‘out‐group’ members 48. This notion is supported by a recent study reporting that oxytocin‐induced noncooperation in the prisoner's dilemma, a trust game, was influenced by fear 49. It was suggested that oxytocin may have increased sensitivity fear‐inducing social stimuli leading to noncooperation 49. Thus, it may be that the previously seen effects of oxytocin on social‐emotional processing are a by‐product of its effects on feelings of belongingness. Further exploration of the effects of oxytocin on expression of emotions in different social boundary conditions may be of interest with respect to evaluating its therapeutic potential.

When viewing the humorous film clip, the AN participants expressed significantly less positive facial affect than the HC participants, although there were no significant differences between the groups when viewing the sad film clip. Relative to the HC group, the AN participants also spend more time looking away from both emotional film clips, and reported experiencing a less positive affect and a more negative affect in response to the humorous and sad film clips. The present findings add to the steady accumulation of evidence indicating that the AN group has difficulties expressing positive emotions and also has a tendency to avoid emotional stimuli 7, 37, 38, 39, 50. Furthermore, previous studies have shown that, relative to HC women, adolescents and adult women with AN do not display more negative facial expressions in response to negative film stimuli despite reporting a more negative affect 38, 51. Taken together, these findings suggest that there may be a general disconnect between subjective experience and external display of emotions and a tendency to avoid emotional engagement. Such difficulties have important affective and social consequences 4, 52 and are thus important targets for interventions.

By contrast to what we expected, AN participants were significantly more accurate than the HC participants with respect to interpreting complex emotions in the RMET. This finding is in direct contrast with previous work showing that AN participants have difficulties with accurately interpreting complex emotions in the RMET 53, 54, 55. However, upon closer inspection, on average, the AN participants in the present study did not appear to outperform those in some previous studies 53, 55. The HC participants, on the other hand, were less accurate than those in previous case–control studies and large sample validation studies 53, 54, 55, 56, 57. Although, it is difficult to know what led to these unexpected findings, it is unlikely to be a result of the HC group having general difficulties with understanding the task. Had that been the case, we would have expected there to be significant differences between the groups in accuracy and response times, which did not occur in the present study. Additionally, the majority of the AN participants (57%) were more familiar with the RMET than HC participants, having completed it before as part of other studies, and some of them performed unexpectedly well on the task, with accuracy percentages of up to 100%. Indeed, once these participants were removed, the differences between the AN and HC groups did not reach significance. However, there still remained a small difference between the groups, with AN participants being more accurate.

Interestingly, we also found significant differences in accuracy and reaction times between sessions 1 and 2 on the RMET across groups, with all participants becoming more accurate and faster in session 2 even though no feedback on performance was given. These findings appear surprising considering that previous test–retest reliability studies have found no such effects in the RMET among healthy populations 58, 59. However, it is of note that, in these studies, a minimum of 2 weeks fell between the first administration of the task and the retest 58, 59, whereas, in the present study, the two sessions were only 1–5 days apart. Other studies that have used shorter intervals between test and retest, ranging from 1 h to 1 week, have found improvements in accuracy and response times in a range of social‐emotional tasks 60, 61. Furthermore, a few studies have found implicit learning effects over the course an emotion recognition task in healthy and other clinical populations, including people with depression and borderline personality disorder 62, 63. Therefore, it is possible that, because the two sessions in the present study were so close, participants became familiarised with the stimuli, leading to a significant improvement in task performance.

The present study also explored differences between participants taking antidepressants and those free of psychotropic medication. As might be expected, those taking antidepressants reported significantly more eating disorder psychopathology, depression and anxiety than those free of psychotropic medication, suggesting that medicated AN participants were more severe. However, when these confounding factors were accounted for, the medicated AN participants remained significantly less accurate with respect to interpreting emotions, and were less expressive and reported a less positive affect when watching the humorous Film 1 than the non‐medicated AN group. Although, it is possible that additional confounders were not accounted for, a recent study investigating facial expressivity among people with AN also reported a reduced expression of positive facial affect among AN participants taking antidepressants 9. Further exploration of the effects of antidepressants on social‐emotional processing in people with acute AN may be of interest.

### Clinical implications

The present findings add further evidence to the wealth of research suggesting that people with AN may have a general disconnect between subjective experience and expression of emotions 38, 51. Appropriate expression of emotions is one of the corner stones of social interaction and a blunted facial affect can have profound affective and social consequences 4, 52. Behavioural studies have found that inhibiting the expression of facial affect in response to emotionally provoking stimuli is associated with increases in blood pressure and reductions in subjective enjoyment 52, 64. Additionally, when viewing videos of people displaying either appropriate, inappropriate or blunted reactions to emotional stimuli, participants rated those people who displayed a blunted response to positive emotional stimuli more negatively and expressed a greater desire for increased social distance from these individuals 4. Thus, these problems with social communication may contribute to the elevated negative mood, isolation and loneliness that are common features of AN 65.

### Limitations

The main limitation of the present study was the relatively short interval between the administration of oxytocin and the first task. However, there are previous studies that have used a similar short interval between administration and the task and have reported significant differences in social‐emotional processing between oxytocin and placebo sessions 66. However, it is not possible to determine whether the lack of oxytocin‐induced effects in the RMET in the present study was not at least partly a result of this short interval. Still, previous work investigating the temporal profile of another neuropeptide similar to oxytocin (i.e. vasopressin) has found that significant effects occur within 10 min of administration 67. Nonetheless, replication of the present findings with a longer interval between administration and tasks is necessary.

Although the sad film has been successfully used in many previous studies 37, 38, 39, in the present study, we found that the majority of the participants were not familiar with the film *Shadowlands* (1999) and did not understand it. Thus, most of the participants were not sufficiently affected by the film and did not express a sufficient negative facial affect to be detected by facereader, which provides a mean valence score for the duration of the film. If participants expressed a negative facial affect on only couple of occasions during the film but were otherwise neutral during the majority of the film, those short expressions would be averaged out in the mean valence score. A previous study used clips from different films tailored specifically for younger participants and found robust results showing reduced facial expressivity in young people with AN when viewing both happy and sad film clips 51. Future research may benefit from tailoring the clips for the participant group to ensure they are sufficiently familiar with the story to engage with the clips.

Compared to previous work 37, 38, 50, 51, the present study employed a modified procedure in which the experimenter stayed in the room during all tasks. Although this allowed the experimenter to tackle some practical difficulties (e.g. a participant moving outside of camera range or watching the clips in a different order), it may have led the participants to feel less able to engage and become involved with the film clips during the film task. Future studies may benefit from finding alternative ways to combat noncompliance, at the same time as giving participants sufficient privacy to engage with the films.

Finally, the present study did not assess participants IQ, which can impact task performance. This is a limitation when comparing the performance of the two groups on the RMET, which requires an understanding of adjectives describing complex emotions. Although this did not directly impact the investigation of the effects of oxytocin in the cross‐over study, future work should conduct a full IQ assessment when attempting to compare the performance of two or more groups on a similar neuropsychological test.

## Conclusions

The present study aimed to investigate the effects of a single dose of intranasal oxytocin on difficulties in the interpretation and expression of emotions in AN. The findings revealed that oxytocin did not significantly influence the interpretation of complex emotions or facial expressivity in response to positive or sad emotional stimuli in AN. These findings are supported by recent work showing that intranasal oxytocin does not improve emotion recognition sensitivity in people with AN and selectively improves the expression of negative emotions in response to threatening stimuli 14, 25, 26, 27. However, replication of these findings with different intervals between oxytocin administration and the task is necessary before firm conclusions can be drawn. Taken together, these findings suggest that there may be anomalies present in social‐emotional processing in people with AN that are not resolved with a single dose of intranasal oxytocin.

## Conflicts of interest

The authors declare that there they have no conflicts of interest.

## Supporting information


**Fig. S1.** Performance on the Reading the Mind in the Eyes (RMET) in the medicated and non‐medicated anorexia nervosa (AN) groups in oxytocin and placebo conditions.

**Fig. S2.** Intensity of expressions of sadness and happiness in response to Film 1 and Film 2 in the medicated and non‐medicated anorexia nervosa (AN) groups in oxytocin and placebo conditions.

**Doc. S1.** Supplementary materials.

**Table S1.** Performance on the Reading the Mind in the Eyes (RMET) in anorexia nervosa (AN) and healthy comparison (HC) participants who had not completed the task before following oxytocin and placebo.

**Table S2.** Accuracy on the Reading the Mind in the Eyes (RMET) by item difficulty following oxytocin and placebo administration in anorexia nervosa (AN) and healthy comparison (HC) groups.

**Table S3.** Performance on the Reading the Mind in the Eyes (RMET) in medicated and non‐medicated anorexia nervosa (AN) participants.

**Table S4.** Expressions of happiness and sadness, and looking away in medicated and non‐medicated anorexia nervosa (AN) participants during Film 1 and Film 2.

**Table S5.** PANAS ratings in medicated and non‐medicated anorexia nervosa (AN) participants following Film 1 and Film 2.

**Table S6.** Correlations between oxytocin‐induced changes in interpretation and expression of emotions and autistic traits, body mass index (BMI) and depression.
