# Supplementary figures and images for: Effects of Intranasal Oxytocin on the Interpretation and Expression of Emotions in Anorexia Nervosa
Source: J Neuroendocrinol. 2017 Mar 8;29(3):n/a. doi: 10.1111/jne.12458 (PMC5363234; doi:10.1111/jne.12458)

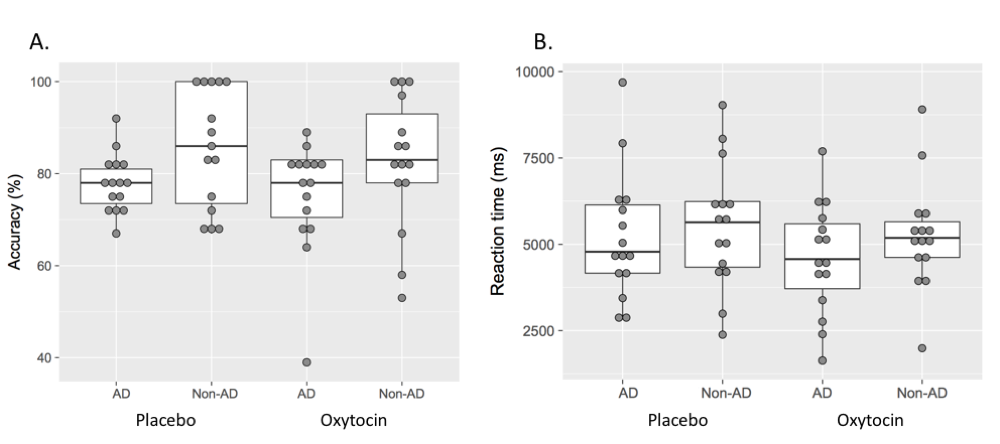

Supplement: Supplementary file 1 — Fig. S1. Performance on the Reading the Mind in the Eyes (RMET) in the medicated and non‐medicated anorexia nervosa (AN) groups in oxytocin and placebo conditions. [file JNE-29-na-s001.png]

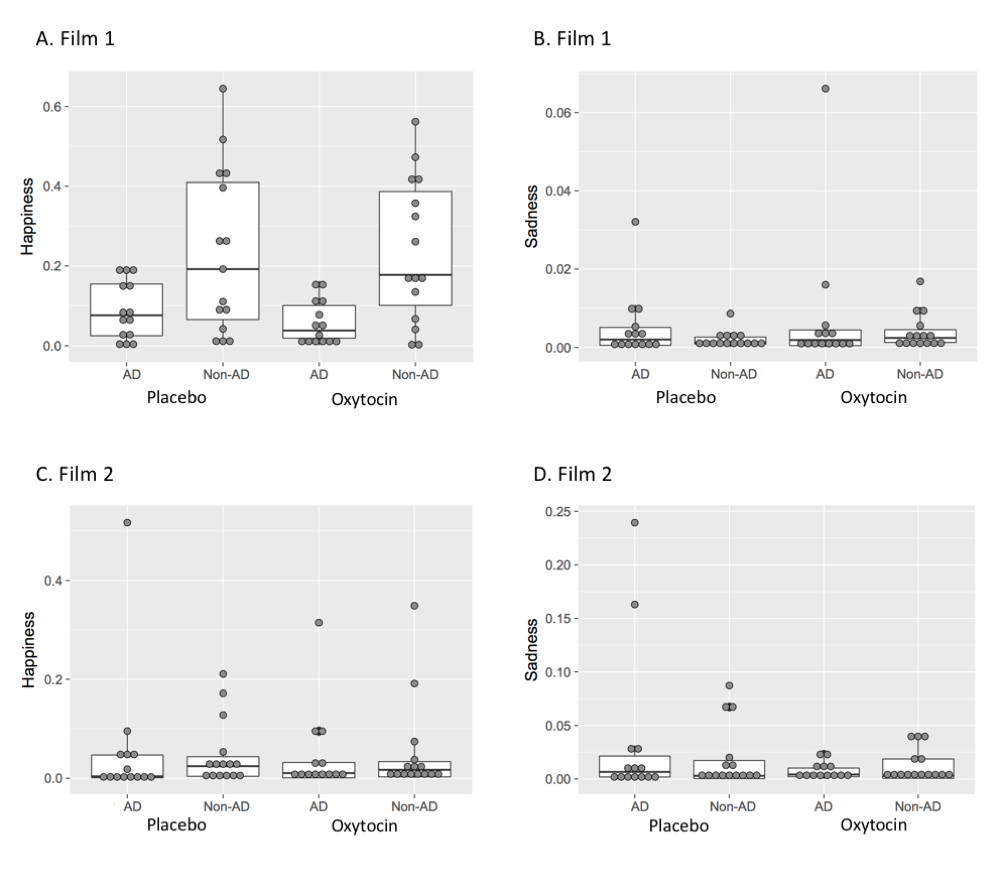

Supplement: Supplementary file 2 — Fig. S2. Intensity of expressions of sadness and happiness in response to Film 1 and Film 2 in the medicated and non‐medicated anorexia nervosa (AN) groups in oxytocin and placebo conditions. [file JNE-29-na-s002.png]
