# Supplementary material for: Effects of Intranasal Oxytocin on the Interpretation and Expression of Emotions in Anorexia Nervosa
Source: J Neuroendocrinol. 2017 Mar 8;29(3):n/a. doi: 10.1111/jne.12458 (PMC5363234; doi:10.1111/jne.12458)
Supplement: Supplementary file 3 — Doc. S1. Supplementary materials. [file JNE-29-na-s003.docx]

# Supplementary materials

## RMET analysis by item difficulty

Drug (oxytocin, placebo) x group (AN, HC) x category (easy, difficult) mixed linear model with 1000 bootstrap repetitions revealed a significant effect of group, with AN participants being significantly more accurate than HCs (Supplementary table 2). As expected there was additionally a significant effect of category with all participants being more accurate in the easy trials than difficult trials. There were no other significant effects or interactions.

## Performance on the RMET in medicated and non-medicated AN participants

Interpretation accuracy and RT in the medicated and non-medicated AN groups following both oxytocin and placebo administration are presented in Supplementary Figure 1. Because the medicated and non-medicated AN groups differed significantly on the EDEQ and DASS an composite score was created adding the total scores together. This composite score was then used to control for differences arising from self-reported psychopathology in all analysis investigating differences between medicated and non-medicated participants.

The results revealed a significant effect of medication on accuracy, with the non-medicated AN participants being significantly more accurate interpreting complex emotions that the medicated AN participants (Supplementary Table 3). There were no other significant effects or interactions influencing accuracy or RT.

## Expressions of happiness and sadness, and looking away in medicated and non-medicated AN participants

Intensity of expressions of sadness and happiness in response to Film 1 and Film 2 following placebo and oxytocin administration are summarised for the medicated and non-medicated AN participants in Supplementary Figure 2 (Film 1: A, B; Film 2: C, D). As above all analyses were conducted controlling for self-reported psychopathology.

As expected, the mixed model revealed only a significant effect of film on expressions of sadness, with all AN participants expressing more sadness while viewing Film 2 than Film 1 (Supplementary Table 4). There was no significant effect of medication status or any other significant main effects not interactions influencing expressions of sadness.

As above, the results also revealed a significant effect of film, with all AN participants expressing more happiness in response to Film 1 than Film 2. Despite controlling for self-reported psychopathology, there was also a significant effects of medication status, with non-medicated AN participants expressing significantly more happiness across films and condition than the medicated AN participants.

Additionally, the results revealed a significant film x medication status interaction. Post-hoc pairwise comparisons revealed that non-medicated AN participants expressed significantly more happiness than medicated AN participants while viewing Film 1 (Z = -4.70, p < 0.001, 95% CI [-0.31, -0.13]), but not Film 2 (Z = -0.38, p = 0.701, 95% CI [-0.10, 0.07]).

Total time medicated and non-medicated AN participants spent looking away from the film stimuli is presented in Supplementary Table 2. There was a significant effect of film with all AN participants spending more time looking away from Film 2 than Film 1. There were no other significant effects of interactions influencing looking away.

## PANAS ratings in medicated and non-medicated AN participants

Both the mixed model exploring ratings of positive affect and that exploring ratings of negative affect found a significant effect of Film (Supplementary Table 5). As above all analyses were conducted controlling for self-reported psychopathology. All AN participants reporting more positive affect in response to Film 1 than Film 2, and more negative affect in response to Film 2 than Film 1.

The mixed model exploring ratings of positive affect revealed a significant effect of group, with non-medicated AN participants reporting significantly more positive affect relative to medicated AN participants (Supplementary Table 5).

## Correlations between oxytocin-induced changes in interpretation and expression of emotions and depression, autistic traits, and BMI,

There were no significant correlations between interpretation or expression of emotions and autistic traits, BMI, or depression within the AN group (Supplementary Table 6).
