# Supplementary material for: Effects of Intranasal Oxytocin on the Interpretation and Expression of Emotions in Anorexia Nervosa
Source: J Neuroendocrinol. 2017 Mar 8;29(3):n/a. doi: 10.1111/jne.12458 (PMC5363234; doi:10.1111/jne.12458)
Supplement: Supplementary file 4 — Table S1. Performance on the Reading the Mind in the Eyes (RMET) in anorexia nervosa (AN) and healthy comparison (HC) participants who had not completed the task before following oxytocin and placebo. [file JNE-29-na-s004.docx]

Supplementary Table 1. Performance on the RMET in AN and HC participants who had not completed the task before following oxytocin and placebo.

|  | Drug | AN (N = 13)  Mean (SD) | HC (N = 29)  Mean (SD) | Χ^2^ statistic, p value |
| --- | --- | --- | --- | --- |
| Accuracy (%) | Oxytocin | 77.08 (7.88) | 69.83 (12.35) | Drug: Χ^2^ = 2.00, p = 0.157  Session: Χ^2^ = 4.09, p = 0.043  Group: Χ^2^ = 2.13, p = 0.145  Drug x Session: Χ^2^ = 1.33, p = 0.249  Drug x Group: Χ^2^ = 0.14, p = 0.706  Session x Group: Χ^2^ = 0.28, p = 0.597  Drug x Session x Group: Χ^2^ = 0.28, p = 0.597 |
|  | Placebo | 74.85 (5.29) | 71.79 (9.63) |  |
| RT | Oxytocin | 5266.14 (1962.82) | 5158.24 (2671.37) | Drug: Χ^2^ = 2.53, p = 0.112  Session: Χ^2^ = 53.55, p < 0.001  Group: Χ^2^ = 0.20, p = 0.651  Drug x Session: Χ^2^ = 3.04, p = 0.081  Drug x Group: Χ^2^ = 5.57, p = 0.018  Session x Group: Χ^2^ = 2.69, p = 0.101  Drug x Session x Group: Χ^2^ = 0.02, p = 0.893 |
|  | Placebo | 4592.68 (1116.02) | 5241.59 (2282.45) |  |

AN = anorexia nervosa, HC = healthy comparison, RMET = Reading the Mind in the Eyes; RT = reaction time
