# Supplementary material for: Effects of Intranasal Oxytocin on the Interpretation and Expression of Emotions in Anorexia Nervosa
Source: J Neuroendocrinol. 2017 Mar 8;29(3):n/a. doi: 10.1111/jne.12458 (PMC5363234; doi:10.1111/jne.12458)
Supplement: Supplementary file 5 — Table S2. Accuracy on the Reading the Mind in the Eyes (RMET) by item difficulty following oxytocin and placebo administration in anorexia nervosa (AN) and healthy comparison (HC) groups. [file JNE-29-na-s005.docx]

Supplementary Table 2. Accuracy on the RMET by item difficulty following oxytocin and placebo administration in AN and HC groups.

|  | Category | Drug | AN (N = 30)  Mean (SD) | HC (N = 29)  Mean (SD) | Χ^2^ statistic, p value |
| --- | --- | --- | --- | --- | --- |
| Accuracy (%) | Easy | Oxytocin | 0.90 (8.30) | 77.20 (13.47) | Drug: Χ^2^ = 0.09, p = 0.763  Category: Χ^2^ = 119.13, p < 0.001  Group: Χ^2^ = 42.11, p < 0.001  Drug x Category: Χ^2^ = 0.09, p = 0.759  Drug x Group: Χ^2^ = 2.47, p = 0.116  Category x Group: Χ^2^ = 0.40, p = 0.526  Drug x Category x Group: Χ^2^ = 1.38, p = 0.240 |
|  |  | Placebo | 85.74 (14.27) | 81.42 (12.88) |  |
|  | Difficult | Oxytocin | 73.52 (16.03) | 62.26 (14.75) |  |
|  |  | Placebo | 71.85 (15.64) | 62.07 (13.20) |  |

AN = anorexia nervosa, HC = healthy comparison, RMET = Reading the Mind in the Eyes
