# Supplementary material for: Effects of Intranasal Oxytocin on the Interpretation and Expression of Emotions in Anorexia Nervosa
Source: J Neuroendocrinol. 2017 Mar 8;29(3):n/a. doi: 10.1111/jne.12458 (PMC5363234; doi:10.1111/jne.12458)
Supplement: Supplementary file 6 — Table S3. Performance on the Reading the Mind in the Eyes (RMET) in medicated and non‐medicated anorexia nervosa (AN) participants. [file JNE-29-na-s006.docx]

Supplementary Table 3. Performance on the RMET in medicated and non-medicated AN participants

|  | Drug | Medicated AN (N = 15) Mean (SD) | Non-medicated (N = 15)  Mean (SD) | Χ^2^ statistic, p value |
| --- | --- | --- | --- | --- |
| Accuracy (%) | Oxytocin | 0.75 (0.12) | 0.83 (0.15) | Drug: Χ^2^ = 1.12, p = 0.290  Medication status: Χ^2^ = 9.30, p = 0.002  Drug x Medication status: Χ^2^ = 0.12, p = 0.734 |
|  | Placebo | 0.78 (0.06) | 0.86 (0.13) |  |
| RT (ms) | Oxytocin | 4599.90 (1614.52) | 5258.73 (1571.47) | Drug: Χ^2^ = 2.42, p = 0.120  Medication status: Χ^2^ = 1.43, p = 0.232  Drug x Medication status: Χ^2^ = 0.29, p = 0.592 |
|  | Placebo | 5221.79 (1843.43) | 5531.64 (1811.55) |  |

All analyses were conducted controlling for self-reported psychopathology (DASS total + EDEQ total). AN = anorexia nervosa; RT = reaction time; RMET = Reading the Mind in the Eyes
