# Supplementary material for: Effects of Intranasal Oxytocin on the Interpretation and Expression of Emotions in Anorexia Nervosa
Source: J Neuroendocrinol. 2017 Mar 8;29(3):n/a. doi: 10.1111/jne.12458 (PMC5363234; doi:10.1111/jne.12458)
Supplement: Supplementary file 7 — Table S4. Expressions of happiness and sadness, and looking away in medicated and non‐medicated anorexia nervosa (AN) participants during Film 1 and Film 2. [file JNE-29-na-s007.docx]

Supplementary Table 4. Expressions of happiness and sadness, and looking away in medicated and non-medicated AN participants during Film 1 and Film 2

|  | Film | Drug | Medicated AN (N = 15) Mean (SD) | Non-medicated (N = 15)  Mean (SD) | Χ^2^ statistic, p value |
| --- | --- | --- | --- | --- | --- |
| Expressions of happiness | Film 1 | Oxytocin | 0.06 (0.05) | 0.24 (0.18) | Drug: Χ^2^ =0.18, p = 0.675  Film: Χ^2^ = 30.79, p < 0.001  Medication status: Χ^2^ = 14.38, p < 0.001  Drug x Film: Χ^2^ = 0.04, p = 0.833  Drug x Medication status: Χ^2^ = 0.19, p = 0.666  Film x Medication status: Χ^2^ = 9.11, p = 0.003  Drug x Film x Medication status: Χ^2^ = 0.28, p = 0.598 |
|  |  | Placebo | 0.09 (0.07) | 0.23 (0.21) |  |
|  | Film 2 | Oxytocin | 0.05 (0.09) | 0.05 (0.10) |  |
|  |  | Placebo | 0.06 (0.14) | 0.05 (0.07) |  |
| Expressions of sadness | Film 1 | Oxytocin | 0.01 (0.02) | 0.004 (0.005) | Drug: Χ^2^ = 0.87, p = 0.351  Film: Χ^2^ = 9.44, p = 0.002  Medication status: Χ^2^ = 0.53, p = 0.465  Drug x Film: Χ^2^ = 2.44, p = 0.119  Drug x Medication status: Χ^2^ = 0.77, p = 0.381  Film x Medication status: Χ^2^ < 0.01, p = 0.954  Drug x Film x Medication status: Χ^2^ = 0.26, p = 0.610 |
|  |  | Placebo | 0.01 (0.01) | 0.002 (0.002) |  |
|  | Film 2 | Oxytocin | 0.01 (0.01) | 0.01 (0.16) |  |
|  |  | Placebo | 0.04 (0.07) | 0.02 (0.03) |  |
| Looking away (seconds) | Film 1 | Oxytocin | 1.14 (3.18) | 1.40 (1.72) | Drug: Χ^2^ = 0.25, p = 0.619  Film: Χ^2^ = 6.01, p = 0.014  Medication status: Χ^2^ = 3.69, p = 0.055  Drug x Film: Χ^2^ < 0.01, p = 0.973  Drug x Medication status: Χ^2^ = 0.99, p = 0.320  Film x Medication status: Χ^2^ = 2.98, p = 0.084  Drug x Film x Medication status: Χ^2^ = 0.63, p = 0.426 |
|  |  | Placebo | 1.00 (1.80) | 1.00 (2.10) |  |
|  | Film 2 | Oxytocin | 1.14 (1.88) | 4.20 (4.43) |  |
|  |  | Placebo | 1.86 (4.11) | 2.87 (5.28) |  |

All analyses were conducted controlling for self-reported psychopathology (DASS total + EDEQ total). AN = anorexia nervosa; Film 1 = humorous film clip; Film 2 = sad film clip
