# Supplementary material for: Effects of Intranasal Oxytocin on the Interpretation and Expression of Emotions in Anorexia Nervosa
Source: J Neuroendocrinol. 2017 Mar 8;29(3):n/a. doi: 10.1111/jne.12458 (PMC5363234; doi:10.1111/jne.12458)
Supplement: Supplementary file 8 — Table S5. PANAS ratings in medicated and non‐medicated anorexia nervosa (AN) participants following Film 1 and Film 2. [file JNE-29-na-s008.docx]

Supplementary Table 5. PANAS ratings in medicated and non-medicated AN participants following Film 1 and Film 2

| PANAS scale | Film | Drug | Medicated AN (N = 15) Mean (SD) | Non-medicated (N = 15)  Mean (SD) | Χ^2^ statistic, p value |
| --- | --- | --- | --- | --- | --- |
| Positive affect | Film 1 | Oxytocin | 10.00 (5.57) | 11.93 (3.58) | Drug: Χ^2^ =0.71, p = 0.400  Film: Χ^2^ = 80.90, p < 0.001  Medication status: Χ^2^ = 8.77, p = 0.003  Drug x Film: Χ^2^ = 0.05, p = 0.824  Drug x Medication status: Χ^2^ = 0.01, p = 0.941  Film x Medication status: Χ^2^ < 0.01, p = 0.996  Drug x Film x Medication status: Χ^2^ = 1.21, p = 0.272 |
|  |  | Placebo | 9.93 (6.20) | 13.40 (4.84) |  |
|  | Film 2 | Oxytocin | 3.31 (2.98) | 6.87 (4.64) |  |
|  |  | Placebo | 4.38 (4.52) | 6.60 (4.60) |  |
| Negative affect | Film 1 | Oxytocin | 4.71 (4.39) | 5.40 (7.47) | Drug: Χ^2^ = 0.37, p = 0.542  Film: Χ^2^ = 25.36, p < 0.001  Medication status: Χ^2^ = 2.06, p = 0.151  Drug x Film: Χ^2^ = 0.14, p = 0.713  Drug x Medication status: Χ^2^ = 1.13, p = 0.287  Film x Medication status: Χ^2^ = 2.65, 0.103  Drug x Film x Medication status: Χ^2^ = 0.02, p = 0.879 |
|  |  | Placebo | 4.93 (3.89) | 3.67 (5.18) |  |
|  | Film 2 | Oxytocin | 7.00 (5.32) | 10.40 (7.65) |  |
|  |  | Placebo | 7.54 (5.49) | 9.47 (5.51) |  |

All analyses were conducted controlling for self-reported psychopathology (DASS total + EDEQ total). AN = anorexia nervosa; PANAS = Positive and negative affect scale; Film 1 = humorous film clip; Film 2 = sad film clip
