# Supplementary material for: Effects of Intranasal Oxytocin on the Interpretation and Expression of Emotions in Anorexia Nervosa
Source: J Neuroendocrinol. 2017 Mar 8;29(3):n/a. doi: 10.1111/jne.12458 (PMC5363234; doi:10.1111/jne.12458)
Supplement: Supplementary file 9 — Table S6. Correlations between oxytocin‐induced changes in interpretation and expression of emotions and autistic traits, body mass index (BMI) and depression. [file JNE-29-na-s009.docx]

Supplementary Table 6. Correlations between oxytocin-induced changes in interpretation and expression of emotions and autistic traits, BMI, and depression

| Task | DASS: Depression | AQ Total | BMI |
| --- | --- | --- | --- |
| RMET: accuracy | ρ = 0.04, p = 0.842 | ρ = -0.18, p = 0.348 | ρ = 0.004, p = 0.983 |
| Film 1: Happiness | ρ = -0.34, p = 0.075 | ρ =-0.07, p = 0.717 | ρ = 0.19, p = 0.339 |
| Film 2: Happiness | ρ = 0.13, p = 0.524 | ρ = 0.24, p = 0.226 | ρ = 0.37, p = 0.050 |
| Film 1: Sadness | ρ = -0.02, p = 0.909 | ρ = 0.08, p = 0.670 | ρ = -0.14, p = 0.488 |
| Film 2: Sadness | ρ = 0.15, p = 0.459 | ρ = -0.10, p = 0.608 | ρ = 0.06, p = 0.764 |

RMET = Reading the Mind in the Eyes; DASS = Depression, Anxiety, and Stress Scale; AQ = Autism quotient; BMI = body mass index
